# Advancements in engineered mesenchymal stem cell exosomes for chronic lung disease treatment

**DOI:** 10.1186/s12967-023-04729-9

**Published:** 2023-12-09

**Authors:** Zhengyao Zhai, Tairong Cui, Jialiang Chen, Xulong Mao, Ting Zhang

**Affiliations:** 1 The First School of Medicine, School of Information and Engineering, The First Affiliated Hospital of Wenzhou Medical University, Wenzhou, 325035 Zhejiang China; 2 Key Laboratory of Heart and Lung, Division of Pulmonary Medicine, The First Affiliated Hospital of Wenzhou Medical University, Wenzhou, 325035 Zhejiang China; 3 Department of Rheumatology, The First Affiliated Hospital of Wenzhou Medical University, Wenzhou, 325035 Zhejiang China

**Keywords:** Mesenchymal stem cell, Engineered exosome, Chronic lung disease, Therapeutic potential

## Abstract

Chronic lung diseases include an array of conditions that impact airways and lung structures, leading to considerable societal burdens. Mesenchymal stem cells (MSCs) and their exosomes (MSC-exos) can be used for cell therapy and exhibit a diverse spectrum of anti-inflammatory, antifibrotic, and immunomodulatory properties. Engineered MSC-exos possesses enhanced capabilities for targeted drug delivery, resulting in more potent targeting effects. Through various engineering modifications, these exosomes can exert many biological effects, resulting in specific therapeutic outcomes for many diseases. Moreover, engineered stem cell exosomes may exhibit an increased capacity to traverse physiological barriers and infiltrate protected lesions, thereby exerting their therapeutic effects. These characteristics render them a promising therapeutic agent for chronic pulmonary diseases. This article discusses and reviews the strategies and mechanisms of engineered MSC-exos in the treatment of chronic respiratory diseases based on many studies to provide new solutions for these diseases.

## Background

Chronic lung diseases include a range of conditions that affect the airways and other lung structures and primarily include chronic obstructive pulmonary disease (COPD), asthma, occupational lung disease, and pulmonary hypertension [1]. Chronic respiratory diseases are a leading cause of death and disability, and an estimated 4 million people succumb to these conditions worldwide each year [2]. Given the significant societal burden of chronic respiratory diseases, there is an urgent need for effective prevention and treatment strategies [3]. Research has demonstrated that mesenchymal stem cells (MSCs) and their exosomes (MSC-exos) can be used for cell therapy and exert a broad spectrum of anti-inflammatory, antifibrotic, and immunomodulatory effects on various human organs and tissues [4]. Engineered MSC-exos possess an enhanced capacity to carry targeted drugs [5], resulting in a more potent targeting effect [6]. Through diverse engineering modifications, these exos can exert different biological effects and produce relatively specific therapeutic outcomes for various diseases. Moreover, engineered stem cell-derived exos may have an increased ability to penetrate physiological barriers and access lesions protected by barriers, thereby exerting therapeutic effects. These advantages position them as a promising treatment option for chronic lung diseases. This article discusses and reviews the strategies and mechanisms of engineered MSC-exos in treating chronic respiratory diseases based on numerous studies and offers new alternatives for addressing these conditions.

## MSCs and MSC-exos

### MSCs

MSCs are among the most extensively studied pluripotent stem cells, have been isolated from various tissues and have the potential to differentiate into osteoblasts, chondrocytes, and adipocytes. As members of the stem cell family, MSCs are easily isolated and cultured, possess self-repair capabilities, and exhibit homing abilities. Additionally, their low heterogeneity and immunomodulatory properties make them a potential therapeutic tool. While the biological characteristics of MSCs confer unique advantages, they also present potential drawbacks. During treatments involving MSCs, the potential risk of tumorigenesis and promoting tumour metastasis should be considered [7, 8]. Furthermore, MSCs carry the risk of activating diffuse intravascular coagulation and may increase the risk of thrombosis through the expression of TF/CD142. In lung diseases, this risk primarily manifests as acute pulmonary thromboembolism. Additionally, due to the multidirectional differentiation potential of MSCs, the instability of MSC traits and phenotypes increases during long-term culture, necessitating more stringent standards in commercialization models [9, 10]. Moreover, MSCs face challenges when penetrating human physiological barriers such as the blood‒brain barrier and the blood‒testis barrier.

Consequently, increasing attention is being focused on cell-free therapy, and MSC-exos have emerged as a promising therapeutic option due to their unique characteristics.

### MSC-exos

MSC-exos are vesicles composed of lipid bilayers measuring 30–120 nm in diameter and containing a diverse array of lipids, proteins, nucleic acids, and cytokines. Exo components can be transferred to other cells, initiating a wide range of cellular signalling pathways and biological responses and serving as crucial tools for intercellular communication. Targeted delivery of exos can prevent nuclease degradation and protect vesicle stability during circulation. Compared to direct donor cell transplantation, exos offer advantages such as ease of production and storage, large-scale preparation, low immunogenicity, and therapeutic effects comparable to those of MSCs. Thus, they play a vital role in delivering membrane-bound proteins, bioactive metabolites, and RNA to recipient cells and are now extensively used to regulate inflammation, wound repair, and vascular recanalization. Considering the characteristics of chronic lung diseases, MSC-exos have some potential advantages. For example, long-term maintenance therapy is critical for chronic lung diseases, and exos from allogeneic MSCs have almost no rejection reaction with T cells, reducing immune rejection during the long-term treatment needed for chronic lung diseases [11]. Chronic lung diseases are also characterized by a complex pathogenesis involving inflammation and airway remodelling. Studies have shown that MSCs and their exos can improve inflammatory responses and resist airway remodelling in various ways [12]. Additionally, during the treatment of chronic lung diseases, the presence of lung biological barriers, such as lung endothelial and epithelial cells, can prevent some therapeutic drugs from achieving maximum efficacy. However, the exos of MSCs can penetrate these barriers, thus ensuring exo concentrations in local tissues and reducing the decrease in effectiveness due to these biological barriers. Finally, evidence-based medicine has shown that MSC-exos have significant potential in treating chronic lung diseases [13].

Conversely, there are limitations to MSC-exo therapy. At present, the main problem facing the use of MSCs-exos is the mass production of exos [14]. Due to the complexity and high demand of the preparation process, exo yield and purity are difficult to ensure. Therefore, increasing the production and purity of exos is imperative to achieving effective treatments and doses. There are already multiple approaches for increasing MSCs and the isolation and purification of exos [14]. A study showed that culturing umbilical cord-derived MSCs in scalable microcarrier-based 3D culture combined with conventional differential ultracentrifugation increased exo production by 20 times compared to that of 2D culture [15]. Moreover, tangential flow filtration (TFF) combined with 3D-MSC cultures increased the production of exos sevenfold compared to the original value [15]. Due to the short circulation half-lives of exos, ensuring the safety and effective dose of exos to treat conditions and maintain therapeutic effects after transplantation pose challenges. Existing research has shown that human blood circulation can rapidly clear exogenously injected exos [16, 17]. As highly bioactive vesicles, exos require stringent storage conditions, which may present obstacles in their use. Current research has revealed that after repeated freezing and thawing at − 80 °C, exos undergo aggregation and fusion, and there is a decrease in the total number of exos and active substances [18]. Furthermore, the use of exos in the human body lacks specific drug targeting indicators, indicating that the therapeutic effect of MSC-exos may be limited and cause side effects. As cellular biological components, the function of MSC-exos is easily influenced by the microenvironment in which they reside. Consequently, there is considerable individual variability in the therapeutic effects on target diseases.

### Engineered MSC-exos

**Fig. 1:**
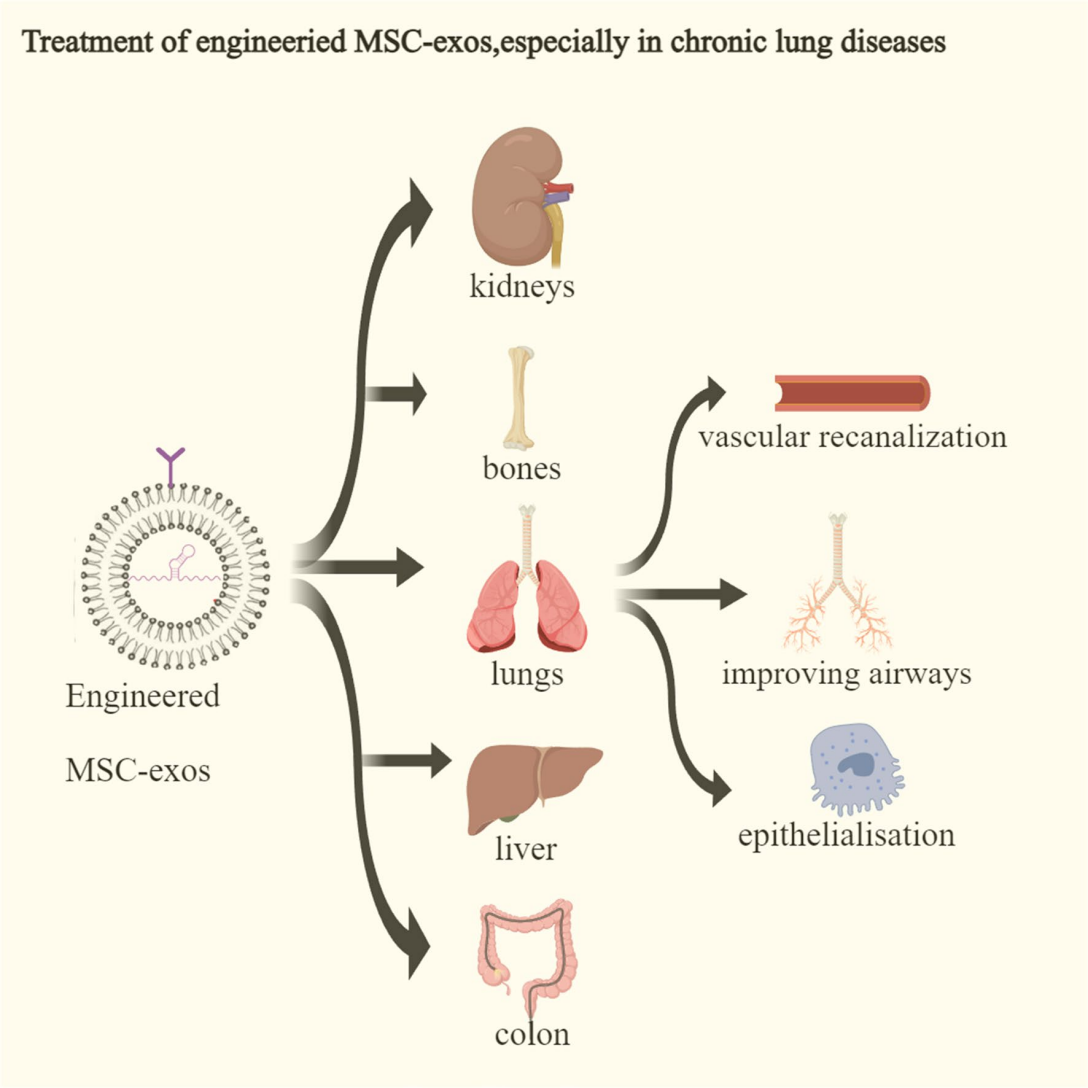
Engineered MSCs exert therapeutic effects on target organs, especially the lungs. Engineered MSC-exos can exert therapeutic effects on organs such as kidneys, bones, lungs, liver, and stomach, and these therapeutic effects can have multiple targets. For example, in the lungs, engineered MSCs can improve the condition of pulmonary blood vessels, airways, and the lung epithelium

Engineered MSC-exos can exert unique biological effects by transporting and delivering bioactive factors (e.g., lipids, proteins, miRNA), demonstrating strong potential in certain aspects of chronic lung disease treatment and prevention (Fig. 1).

**Fig. 2:**
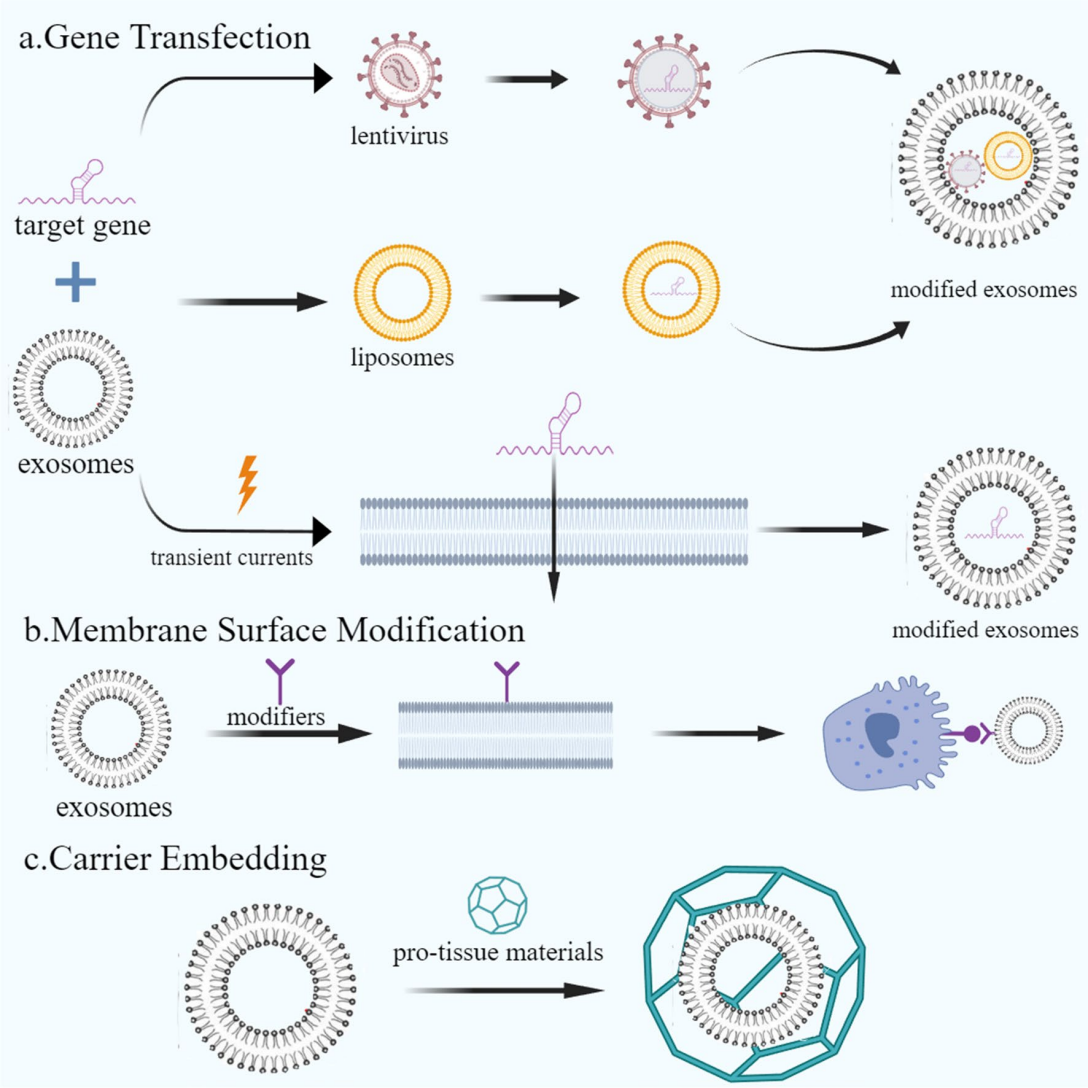
MSC engineering methods. The engineering methods of MSCs can be roughly divided into gene transfection, surface modification, and carrier embedding. Among them, gene transfection is represented by lentiviral transfection and liposome transfection, surface modification mostly uses specific molecular modifiers, and there are carrier embedding such as hydrogels, which are commonly used. Engineering MSCs has unique advantages, enhance targeting, can cross physiological barriers, and exhibit stability in the local microenvironment. These methods can also be combined to improve the capabilities of engineered MSCs

MSC-exos hold vast engineering potential. Engineering modifications can be made to factors such as vesicle content, vesicle membrane components, and transport media, and the action target can be modified to enhance their biological properties in various ways, thereby expanding their potential as a means of disease treatment (Fig. 2). Research has demonstrated that many engineering approaches can be used to modify the homing peptides or ligands on the surface of exos, conferring highly specific targeting capabilities to exos and consequently enhancing their therapeutic efficacy [19]. Different methods for the preparation of engineered MSCS-exos are outlined below.

### Viral and nonviral transfection

#### Virus-mediated gene transfer

Virus-mediated gene transfer engineering is one of the more mature modification techniques in the field of MSC-exo engineering. The gene components delivered by the virus can be stably and efficiently expressed in exos. In the classic viral gene delivery method, transfection using a lentivirus as a vector offers numerous advantages, such as significantly improving transfection stability and reducing the risk of cancer, thereby promoting the therapeutic response and enhancing the therapeutic effect [20–22]. Other studies have shown that lentivirus-transfected MSC-exos exhibit low immunogenicity, and lentivirus transfection is conducive to constructing stable cell lines, thereby reducing the occurrence of target gene damage [23, 24]. For example, Han et al. constructed EPO gene-modified MSCs and MSC-exos using a lentiviral vector that effectively inhibited airway remodelling in an albumin-induced chronic asthmatic mouse model [25]. However, retroviruses directly integrate the target gene into the cell chromosome, which may cause damage to the original gene [26]. Moreover, the production cost is high, which is not conducive to large-scale stable production [27, 28].

#### Nonviral pathway-mediated gene transfer

Nonviral pathway-mediated gene transfer can be primarily divided into electroporation, liposomes, and nanomaterials (carrier or polymer). Compared to viral transfection, these processes are associated with lower immunogenicity, lower production costs, higher efficiency, and better biosafety and flexibility [29, 30], and they are widely used. Electroporation uses transient currents to reversibly open the lipid bilayer, thereby delivering nucleic acids and other substances into exos. Due to its short opening time, it hardly causes damage to exos, rendering it a highly efficient and convenient method [5, 31]. Depending on the initial location of the genetic material being transferred, there are two methods. The first method directly electroporates exos to transfer genetic material into exos. Han et al. used a lentivirus to construct fusion gene iRGD-Lamp2b-modified MSCs, isolated and purified the exos, and loaded anti-miRNA-221 oligonucleotides (AMO) into exos by electroporation, effectively inhibiting the proliferation of colon cancer cells in vitro [32]. The second method directly transfers genetic material into MSCs and indirectly obtains engineered exos that are secreted by MSCs. Katakowski et al. electroporated MSCs to transfect plasmid DNA and then further extracted exos for subsequent experiments [33]. Liposomes and exo membranes with similar solubilities and high biocompatibility [30] can also be used to deliver related substances [6]. Fan et al. used liposome-transfected microRNA-146a-rich engineered MSC-exos, which showed therapeutic effects on diabetic peripheral neuropathy [34]. Nanomaterials are mostly biodegradable polymer nanoparticles (NPs), which are internalized by exos or adsorbed on their surfaces through endocytosis. With their safety, unique physical properties [35] and medicinal value, NPs have attracted widespread attention. Exos loaded with magnetic NPs (such as Fe_3_O_4_) enter the body, and an external static magnetic field (SMF) can attract exos to the site; this method is typically used to increase wound healing and bone regeneration [36].

#### Membrane surface modification

Surface modification techniques, which covalently or noncovalently bind overexpressed proteins in diseased or damaged tissues, can increase the number of MSCs transported to the target and potentially enhance subsequent therapeutic effects [37]. Surface modifications can be easily adjusted to alter or improve their biochemical and physicochemical properties [38]. Covalent modification involves the covalent binding of modifiers to molecules on the exo membrane, thereby enhancing their targeting capabilities [39, 40]. Alendronate (ALN) possesses an affinity for bone surfaces and has osteogenic properties [41] and can be used for bone-targeted drug delivery [42]. Zheng et al. used ALN-coupled polyethylene glycol-modified phospholipid (DSPE-PEG-ALN) to surface-modify platelet lysate-derived exos (PL-exos) and discovered that PL-exo-ALN effectively accumulated on the bone surface, thereby promoting osteogenic differentiation [43]. Noncovalent binding uses ligand‒receptor binding, electrostatic effects, hydrophobic interactions, and nucleic acid aptamer modifications to achieve specific binding to different organs [5, 44, 45]. Patrick et al. used microgel crosslinking to deplete lysine residues with positively charged amines, thereby achieving a net negative charge on crosslinked peptides and isolating biomolecules with positively charged domains. MSCs were cultured on the surface of these microgels, which were isolated from cytokines on the core matrix, prolonging the immunomodulatory phenotype of mesenchymal matrix cells [46–48]. Han et al. identified RUNX2 as a direct target of miR-221 and used an aptamer delivery system to deliver normal BMSC-exos to BMSCs, thereby inhibiting the bone formation caused by the high expression of miR-221 in diabetic mouse exos, reducing bone marrow fat accumulation, and enhancing bone regeneration [49].

#### Carrier embedding

In tissue repair and regenerative medicine, exos often need to be embedded with pro-tissue materials. Hydrogels typically contain large molecules such as polysaccharides, hyaluronic acid, sodium alginate, and polyethylene glycol [50–53], which are compatible with tissue and exhibit properties such as water absorption, buffering, mechanical firmness, and large pore sizes to accommodate and embed exos. This allows exos to release drugs and bioactive substances while being degraded during tissue repair [52]. Zhou et al. encapsulated hADSC-exos in PF-127 hydrogel and demonstrated that the hADSC-exos/PF-127 combination promoted wound healing and cell proliferation in mice, enhanced angiogenesis and collagen synthesis, and accelerated re-epithelialization by slowly releasing exos, thereby reducing the frequency of administration [54]. In recent years, 3D bioprinting technology has been extensively used for biological tissue repair and regeneration due to its ability to customize the shape and structure of biological materials. Bioprinting can print living cells, bioactive substances, and tissue materials and assemble bioactive structures; using hydrogels and other pro-tissue materials as bio-inks, bioactive substances such as drugs, exos, and growth factors can be embedded in them, and these substances can be used to regenerate tissues and organs, particularly bone regeneration [55–58]. Wang et al. used a hybrid hydrogel system as a bio-ink, in which thermosensitive gelatine methacrylate (GelMA) served as the basis of the hybrid hydrogel, and methacrylic acid silk (SilMA) was added to improve the release of exos. The study emphasized the pivotal role of SC-exos in regulating SCs in the bone regeneration microenvironment, which could enhance the bone regeneration microenvironment and promote bone repair [57].

## Chronic lung diseases

### COPD

COPD is a prevalent, preventable, and treatable disease characterized by persistent airflow limitation. This limitation progressively worsens and is closely associated with the chronic inflammatory response of the airways and lungs to toxic particles or gases, chronic bronchitis, and emphysema. COPD can further develop into pulmonary heart disease and respiratory failure, potentially leading to death [12]. Previous studies have preliminarily identified the cell communication pathway between damaged type 2 alveolar epithelium and MSCs mediated by exosomal lncRNA TCONS_00064356 in the pathological process of COPD. This pathway is beneficial for the proliferation and migration of MSCs and enhances mitochondrial synthesis and transfer [59]. The role of MSCs and their exos in COPD treatment has been preliminarily investigated. It has been demonstrated that human adipose-derived MSCs can differentiate into alveolar epithelial cells through mesenchymal–epithelial transformation, thereby improving the ventilation function indicators of emphysema mouse models. This suggests the potential for ameliorating the ventilation barrier of COPD [60]. Another study confirmed that the transplantation of human umbilical cord MSCs (hUC-MSCs) and their exos could treat the loss of the alveolar septum in the lungs of rats with emphysema and reduce the levels of the NF-κB subunit p65 in tissue, thereby exerting a protective effect on rats with emphysema [61]. COPD has a risk of acute exacerbation, which often becomes the primary factor that threatens the lives of COPD patients. MSCs can protect damaged lung cells by promoting mitochondrial transfer, reducing mitochondrial dysfunction and cell apoptosis induced by acute oxidative stress in damaged lung cells [62]. Similarly, it has been proven that MSCs and their exos can inhibit cigarette smoke-induced lung inflammation and injury in mouse models by promoting mitochondrial transfer, thereby protecting mitochondrial respiration in mouse bronchial epithelial cells. This suggests that MSCs and their exos protect against bronchial epithelial damage caused by toxic smoke [62]. Although there is no clear use of engineered MSC exos in the field of COPD, considering the anti-inflammatory and protective effects of MSCs and their exos on multiple animal pathological models, we believe that engineered MSC exos have therapeutic potential in this disease field.

### Asthma

Asthma is a chronic inflammatory disease characterized by airway hyperresponsiveness and episodic airflow obstruction that is closely related to environmental allergens and exhibits significant individual differences. The current mainstream treatment strategies for asthma primarily include corticosteroids and long-acting and short-acting β-receptor agonists (LABA, SABA), but their effects on preventing asthma recurrence and severe asthma are still limited. Therefore, an efficient, safe, and targeted treatment method is urgently needed. Engineered MSC-exos can carry therapeutic biological factors and specific components to aid in the prevention and treatment of asthma. Studies have shown that engineered MSC-exos loaded with ovalbumin (OVA) can reduce the levels of IgE and IL-4 in sensitized BALB/c mice while increasing the levels of IFN-γ and TGF-β and reducing eosinophil counts [63]. Engineered MSC-exos not only play a role in asthma prevention but also regulate abnormal airway smooth muscle cells during by carrying and delivering different molecules. Studies have shown that engineered adipose-derived MSC-exos carrying miR-301a-3p could target and promote STAT3 expression, reverse platelet-derived growth factor-BB (PDGF-BB)-induced airway smooth muscle cell proliferation and migration, induce airway smooth muscle cell apoptosis, and reduce the secretion of inflammatory factors [64]. Another study showed that engineered human bone marrow-derived MSCs carrying microRNA-188 could inhibit the JARID2/Wnt/β-catenin axis, thereby reducing inflammatory cell infiltration, mucus production, and collagen deposition in the lung tissue of asthmatic mice [65].

In addition to the role of engineered MSC-exos in asthma prevention and the regulation of abnormal airway smooth muscle cells in asthma, these factors have also been proven to have direct anti-inflammatory effects. Studies have shown that human MSCs transfected with a miR-138-5p inhibitor activated SIRT1 and inhibited the HMGB1/TLR4 pathway in an OVA-induced asthma syndrome mouse model, and the experiment proved that the inflammatory response in mice was weakened, as determined by the levels of histamine, IgG, TNF-α, and IL-6 [66].

### Pulmonary fibrosis

Pulmonary fibrosis is a progressive, chronic fibrotic interstitial lung disease with a poor prognosis that ultimately leads to respiratory failure and death. The pathogenesis remains unclear, resulting in a lack of targeted and effective treatment methods in clinical practice. Engineered MSC-exos are easily expanded and prepared, not readily rejected by the immune system, and can efficiently transport and deliver targeted molecular substances into lung tissue cells. This makes them a potentially effective treatment for lung tissue cells damaged by fibrosis. Engineered MSC-derived extracellular vesicles (with exos as their primary active components) have direct antifibrotic effects on the lung. Research has confirmed that in a silica-induced pulmonary fibrosis model, human umbilical cord MSC-derived extracellular vesicles (HucMSCs-EVs) could transfer miR-223-3p to alleviate pulmonary fibrosis by inhibiting the circPWWP2A/miR-223-3p/NLRP3 axis. HucMSCs-EVs have also been proven to reduce the macrophage-mediated inflammatory response, limit fibroblast activation and proliferation, decrease the secretion of inflammatory factors (NLRP3, IL-1β, IL-18, and cleaved caspase-1), and alleviate the deposition of fibrosis-related factors (collagen I, collagen III, fibronectin, and α-SMA), thereby regulating lung function [67]. Engineered MSC-derived extracellular vesicles can also inhibit the biological activity of fibroblasts by carrying bioactive molecules, thereby alleviating the development of pulmonary fibrosis on a macroscopic level. Studies have shown that bone marrow MSC-derived extracellular vesicles (BMSC-EVs) can transport and deliver miR-186 to downregulate the expression of SOX4 and DKK1, thereby blocking fibroblast activation and ultimately alleviating pulmonary fibrosis [68]. Another study confirmed that BMSC-derived extracellular vesicles overexpressing miR-29b-3p could downregulate FZD6 expression, ultimately inhibiting the proliferation, migration, invasion, and differentiation of lung interstitial fibroblasts at the epigenetic level [69]. Engineered MSC-exos can also alleviate lung epithelial-mesenchymal transition (EMT) during pulmonary fibrosis. In related studies, modified HucMSCs-EVs expressing the miR-26a-5p lentivirus could inhibit the Adam17/Notch signalling pathway in MLE-12 cells, thereby improving EMT in silica-induced pulmonary fibrosis [70]. This suggests that engineered MSCs can affect pulmonary fibrosis by inhibiting cell transformation.

### Lung cancer

Lung cancer is the leading cause of cancer-related death worldwide. The two main forms of lung cancer are non-small cell lung cancer (NSCLC) (accounting for approximately 85% of all lung cancers) and small cell lung cancer (approximately 15%). Although progress has been made in early detection and standard treatment, non-small cell lung cancer is often diagnosed at an advanced stage and has a poor prognosis. The treatment and prevention of lung cancer are major unmet needs, and a low-risk, efficient, and targeted treatment method is urgently needed [71]. Engineered MSC-exos can serve as effective biological carriers that exert regulatory effects on the malignant behaviours of lung cancer cells, including proliferation, migration, invasion, and metastasis. Studies have shown that in NSCLC cell models and animal models, engineered MSC-derived exo-mediated miR-631 delivery could control NSCLC malignant behaviours by regulating the transcription factor 2/phosphatidylinositol 3-kinase/Akt signalling pathway [72]. Engineered MSC-exos transport and deliver miR-204 to act on NSCLC cells, and the overexpression of miR-204 inhibits KLF7 expression and AKT/HIF-1α pathway activity, thereby inhibiting NSCLC migration and invasion [73]. Bone marrow-derived MSCs (BMSCs) can produce miR-126-3p to target and inhibit CCR1 expression, thereby inhibiting neural cadherin (N-cadherin, N-cad) and vimentin expression, promoting epithelial cadherin (E-cadherin, E-cad) expression, and ultimately inhibiting the proliferation, migration, and invasion of A549 lung cancer cells [74]. Engineered MSC-exos can also transport and deliver negative regulatory factors, intensifying the malignancy of tumours. Studies have shown that human bone marrow-derived MSC-exos (BMSC-exos) can deliver miR-425 into lung cancer cells, inhibit CPEB1 expression, and promote lung cancer cell proliferation, invasion, and metastasis [75]. Furthermore, studies have shown that human BMSC-exos can mediate E2F2 expression by delivering miR-631 to NSCLC cells to regulate NSCLC malignant behaviours [76]. Considering the relationship between malignant lung cancer behaviours and their internal death outcomes, engineered MSCs may be a treatment that promotes programmed death in tumour cells, but their safety and effectiveness still need to be considered. Engineered MSC-exos can also exert various effects depending on their preculture conditions. Studies have shown that unstimulated human bone marrow-derived MSC-exos carrying miR-21-5p act on A549 and H23 lung cancer cells, downregulating PTEN, PDCD4, and RECK gene expression and promoting proliferation, survival, invasion, EMT, and macrophage M2 polarization. Notably, after hypoxic pretreatment, the effects of engineered MSC-exos were more significant [77].

### Pulmonary arterial hypertension (PAH)

PAH is a chronic lung disease characterised by pulmonary artery remodelling [78] that often results from respiratory system lesions. It is characterized by persistent pulmonary arterial hypertension, which can progress to right heart failure and death as the disease advances. Due to the chronic and progressive nature of PAH, preventing and reversing its occurrence and development are critical. MSCs exerts therapeutic effects by altering the gut microbiota in mice with PAH [79]. Additionally, MSCs have been shown to attenuate hypoxia-induced PAH by activating the P53 and NF-kB signalling pathways via TNF-α [80]. Considering their biological characteristics, MSC-exos may exert robust therapeutic effects. Existing research reveals that exos from Wharton’s jelly-derived MSCs (WJ-MSCs) improved vascular density and reduced pulmonary artery pressure in a hyperoxia rat model, thereby inhibiting ventricular remodelling [81]. This suggests the benign induction of MSC-exos during lung development. MSC-exos also play a role in targeting miRNAs. In the study by Li et al., MSC-NV intervention improved PAH lesions induced by colchicine in rats, and by analysing and knocking out their internal genetic material, the possible use of miR-125b-5p and miR-100-5p as therapeutic genetic material was clarified to some extent [82]. MSC-exos can also block vascular remodelling in PAH by regulating the Wnt5a/BMP signalling pathway [83]. Furthermore, research has confirmed that MSC-exos can upregulate the expression of Wnt5a in pulmonary vascular cells of hypoxic rat models, providing a possibility for clinical treatment [84]. Bronchopulmonary dysplasia is a component of PAH and a common chronic lung disease in preterm infants. Numerous studies have demonstrated the therapeutic effects of MSCs and their exos on bronchopulmonary dysplasia and its complications [85–89]. Engineered MSC-exos have been examined, and existing research shows that ReNcell-EVs (a type of exo derived from human neural stem cell lines) coupled with the CAR (CARSKNKDC) peptide (a peptide identified by bioorthogonal chemistry as specifically targeting hypertensive pulmonary arteries) could deliver the endogenous and highly expressed miRNAs let-7b-5p, miR-92b-3p, and miR-100-5p to inhibit the proliferation, migration, and phenotypic transformation of hypoxia-induced pulmonary artery smooth muscle cells and suppress microvascular endothelial cell apoptosis and the mutual transformation between endothelial cells and mesenchymal cells, thereby achieving effective targeted therapy [90]. The engineering of MSC-exos, which has been proven effective in PAH, is still limited, but a comparison of horizontal and vertical analyses reveals the potential for the great therapeutic value of engineered MSC-exos.

## Conclusions and prospects

Chronic lung diseases often have complex pathogeneses and atypical clinical manifestations, and due to their chronic nature, they emphasize the importance of reversing the disease and maintaining long-term treatment outcomes. Conventional drug treatments have limited effects, mainly alleviate the disease rather than reversing it, and cannot achieve satisfactory medical results. MSC-exos, which are an emerging treatment, have received extensive attention in recent years, but due to the pharmacokinetic characteristics of exos themselves, their prospects in chronic lung diseases are limited. However, engineered exos have highly specific molecular targeting characteristics and can intervene in the occurrence and development of chronic lung diseases through specific protein binding and participating the activation or inhibition of signalling pathways. This largely compensates for the defects of MSC-exos such as fast metabolism and the lack of specific targeting markers in the human body. Moreover, due to low immunogenicity, large-scale preparation, and the storage of exos, the safety and feasibility of the long-term treatment of chronic lung diseases are guaranteed to some extent. To date, most of the research on the treatment of chronic lung diseases with engineered MSC-exos has been in the basic experimental stage. As an emerging treatment strategy, its safety, effectiveness, and feasibility still need further evaluation, and so high-stage clinical trials are urgently needed. In addition, since engineered MSC-exos can obtain different therapeutic characteristics through different engineering methods, their heterogeneity in different patients can result in good targeting adaptability, providing new ideas for the individualized treatment of chronic pulmonary diseases. Although engineered stem cells are an emerging treatment strategy and require further research and clinical trials, their enormous potential does suggest the possibility for conquering difficult-to-treat pulmonary diseases.

## Data Availability

Not applicable.

## Abbreviations

MSCs: Mesenchymal stem cells
MSC-exos: Exosomes
COPD: Chronic obstructive pulmonary disease
miRNA: MicroRNA
AMO: Anti-miRNA-221 oligonucleotides
DNA: Deoxyribonucleic acid
NPs: Nanoparticles
SMF: Static magnetic field
ALN: Alendronate
DSPE-PEG-ALN: ALN-coupled polyethylene glycol-modified phospholipid
PL-exos: Platelet lysate-derived exosomes
hADSC-exos: Human adipose-derived stem cells derived exosomes
3D: 3 Dimensions
GelMA: Gelatin methacrylate
SilMA: Methacrylic acid silk
SC-exos: Stem cell-derived exosomes
lncRNA: Long non-coding RNA
hUC-MSCs: Human umbilical cord MSCs
LABA, SABA: Long-acting and short-acting β-receptor agonists
OVA: Ovalbumin
IgE: Immunoglobulin E
IL-4: Interleutin-4
IFN-γ: Interferon gamma
TGF-β: Transforming growth factor-β
PDGF-BB: Platelet-derived growth factor-BB
IgG: Immunoglobulin E
TNF-α: Transforming growth factor-α
IL-6: Interleutin-6
HucMSCs-EVs: Human umbilical cord mesenchymal stem cell-derived extracellular vesicles
BMSCs: Bone marrow-derived mesenchymal stem cells
BMSC-EVs: Bone marrow mesenchymal stem cell-derived extracellular vesicles
IL-1β: Interleutin-1β
IL-18: Interleutin-18
HBEC EV miRNA: Human bronchial epithelial cell-derived extracellular vesicles carrying microRNA
NSCLC: Non-small cell lung cancer
N-cadherin, N-cad: Neural cadherin
E-cadherin, E-cad: Epithelial cadherin
BMSC-exos: Human bone marrow-derived mesenchymal stem cell-derived exosomes
WJ-MSCs: Wharton’s jelly mesenchymal stem cells
